# Edwin Chadwick: A Pioneer of Public Health Reform and His Role in Sanitary Awakening

**DOI:** 10.7759/cureus.68858

**Published:** 2024-09-07

**Authors:** Pooja Mary Vaishali, Nisha Boopathy

**Affiliations:** 1 Department of Community Medicine, Saveetha Medical College and Hospital, Saveetha Institute of Medical and Technical Sciences, Saveetha University, Chennai, IND

**Keywords:** biographies, edwin chadwick, historical vignette, medical innovation, medical stories, sanitary awakening

## Abstract

Edwin Chadwick (1800-1890) was a central figure in the 19th-century public health reform movement in Britain. His work was instrumental in the sanitary awakening, a movement that revolutionized public health through the systematic improvement of urban sanitation and hygiene. As a lawyer by training, Chadwick was deeply influenced by Jeremy Bentham's welfare maximization theory, which emphasized the greatest good for the greatest number. His most significant contribution was his 1842 publication, "The Report on the Sanitary Condition of the Labouring Population," in which he documented the deplorable conditions faced by the working class and the link between poor sanitation and disease. Chadwick's advocacy led to the passage of the Public Health Act of 1848, which established local health boards and marked the beginning of modern public health systems. His focus on clean water, efficient sewage systems, and waste management not only reduced the spread of diseases like cholera and typhoid but also set the stage for future public health initiatives globally. The sanitary awakening, largely driven by Chadwick's efforts, highlighted the critical connection between environment and health, a principle that continues to underpin public health practices today. Chadwick's legacy lives on in the ongoing efforts to improve urban living conditions and prevent disease through public health infrastructure.

## Introduction and background

Edwin Chadwick was born in Manchester on 24 January 1800. His father encouraged him to read books by radicals such as Tom Paine. Chadwick went to London to study law, but his personal finances were limited. He made money by writing essays for publications such as the ‘Westminster Review’. Despite his training in law, his essays were usually on scientific principles and how they could be applied in democratic government. His essays attracted the attention of Jeremy Bentham, who employed Chadwick as his literary assistant and left him a large sum of money in his will. His formative education was characterized by a keen interest in the natural sciences, which later expanded to encompass the social sciences under the tutelage of utilitarian thinkers such as Jeremy Bentham. Although he initially pursued a career-in-law, Chadwick became increasingly involved in public administration and social reform, driven by his unwavering belief in the welfare maximization principle of achieving the greatest good for the greatest number. His career trajectory took a significant turn when he was appointed to the Poor Law Commission in 1832, a position that afforded him the opportunity to observe firsthand the deplorable living conditions of the working poor in urban areas [1]. These experiences ignited his commitment to public health reform, particularly sanitation enhancement. Chadwick's most notable contribution was the publication of "The Report on the Sanitary Condition of the Labouring Population" in 1842, a groundbreaking document that thoroughly detailed the correlation between substandard living conditions, particularly inadequate sanitation, and the prevalence of disease [2].

This review encompasses several crucial aspects of Edwin Chadwick's contribution to public health reform and sanitary awakening. First, it provides an in-depth analysis of Chadwick's seminal report, "The Sanitary Report of 1842," including its methodology, findings, and recommendations, as well as its immediate impact on public health policies in Britain. Second, the review explores Chadwick's role in advocating for and securing the passage of the Public Health Act of 1848 and other related reforms, emphasizing his commitment to government intervention in public health [3]. Third, it examines how Chadwick's work influenced the design and implementation of critical public health infrastructure, such as sewage systems and water supply networks. Fourth, the review discusses the controversies and criticisms Chadwick faced, both during his lifetime and in modern evaluations, particularly regarding his methods and the social implications of his reforms. Finally, it will assess the long-term impact and legacy of Chadwick's work, exploring how it not only catalyzed the sanitary awakening of the 19th century but also established enduring principles that continue to guide public health initiatives today. This thorough review demonstrates Chadwick's lasting influence on global public health practices and its significance as a pioneer in the field.

## Review

Sanitary awakening 

**Figure 1:**
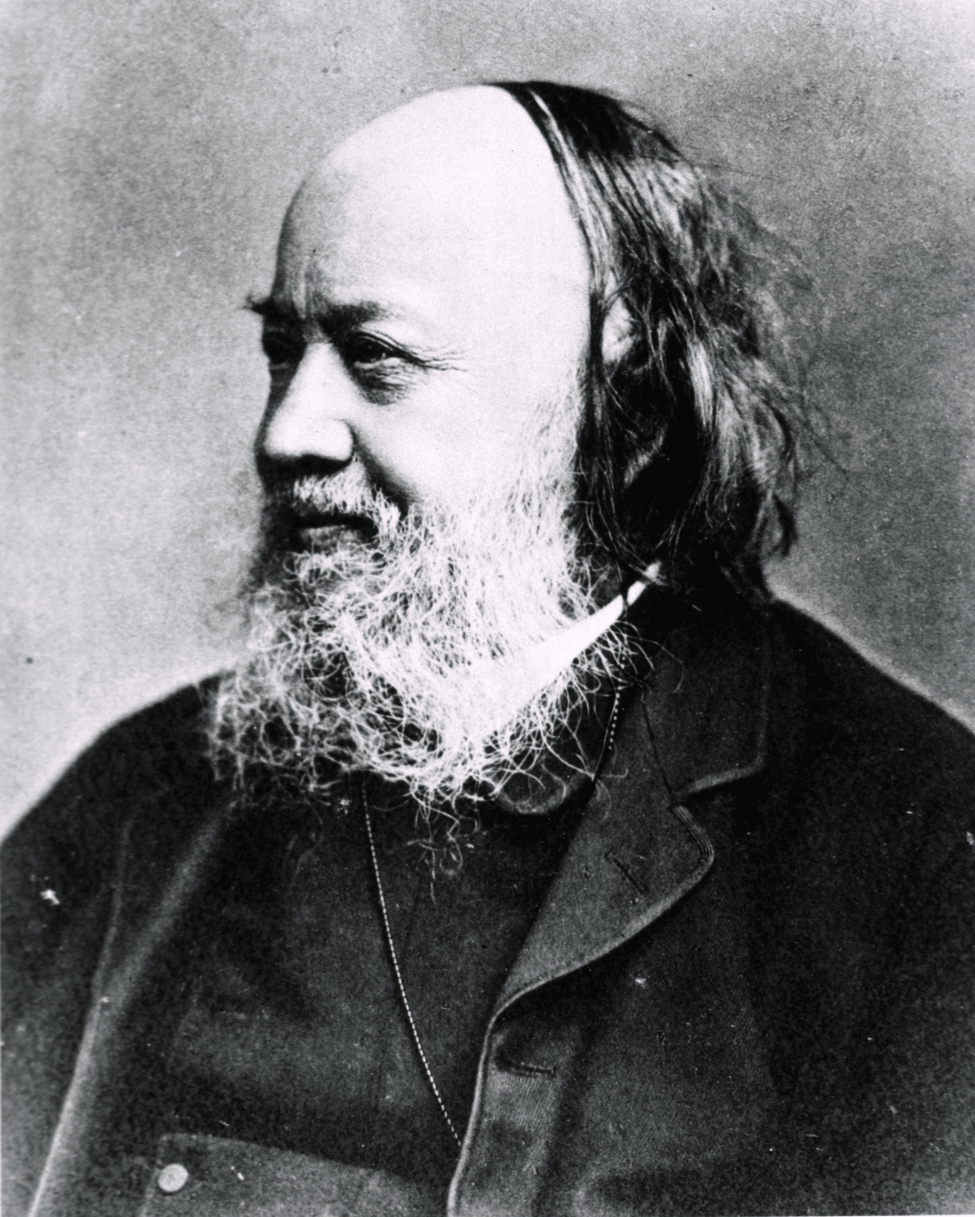
Images from the History of Medicine (IHM) Source: National Library of Medicine (NLM) [6]; NLM Image ID: B08321

Chadwick's work significantly influenced the history of public health, particularly during the 19th-century sanitary awakening (Figure 1). This movement was largely driven by Chadwick's findings and advocacy, which highlighted the essential role of sanitation in preventing diseases and promoting public health. His report not only revealed the adverse health effects of inadequate sanitation but also presented a convincing evidence-based argument for government intervention in this area. Consequently, the Public Health Act of 1848 was passed, establishing the foundation for modern public health systems, including the formation of local health boards and the construction of critical infrastructure, such as sewage systems and clean water supplies [4]. Chadwick's influence extended beyond his lifetime, paving the way for the development of public health policies that continue to shape contemporary practice. His focus on the connection between the environment and health remains relevant today, as public health officials confront the challenges of urbanization, pollution, and disease prevention. Sanitary awakening, sparked by Chadwick's efforts, was not merely a response to the immediate public health crises of the 19th century but a transformative movement that fundamentally altered the way societies perceive and manage public health [5].

Before the mid-19th century, the state of public health and sanitation in Britain was deplorable, especially in rapidly industrialized urban areas. The Industrial Revolution has resulted in unprecedented population growth in cities, leading to overcrowded living conditions, inadequate housing, and poor infrastructure. Many working-class families are compelled to reside in poorly ventilated, damp, and unsanitary dwellings with minimal access to clean water or proper sewage disposal. These circumstances have facilitated the rapid spread of infectious diseases, which has catastrophic consequences. In the early 1800s, the absence of organized waste management systems led to human and animal waste accumulating in the streets or being dumped into rivers, which were also utilized as sources of drinking water. The heavily polluted River Thames, for instance, significantly contributed to the high incidence of waterborne diseases, such as cholera, dysentery, and typhoid fever [7]. The occurrence of these diseases is particularly common in urban regions, and recurring outbreaks often lead to the loss of numerous lives. For instance, the cholera outbreak in London in 1832 resulted in more than 6,000 fatalities within a few months, emphasizing the severe health risks associated with substandard sanitation conditions. In urban areas, the mortality rate is significantly higher than that in rural regions, primarily due to unsanitary living conditions [8]. In fact, in 1838, the estimated life expectancy for a laborer in London was only 17 years, as opposed to 38 years in rural England, indicating a pressing need for comprehensive public health reforms. The living conditions of the working poor were notably depleted, with exceptionally high rates of infant mortality and recurrent outbreaks of diseases, such as tuberculosis, smallpox, and scarlet fever. The absence of clean drinking water and the lack of effective sewage disposal systems have exacerbated the spread of these diseases, resulting in a public health crisis that poses a threat to the entire population.

Sanitation reform

The public health situation in Britain was in dire need of improvement, prompting a push for sanitation reform. In 1832, Prime Minister Earl Grey initiated the Royal Commission of Enquiry on Poor Law. Chadwick's growing reputation led to his appointment as an assistant commissioner tasked with gathering data from the Commission. His writing prowess was used to compose approximately one-third of the final report, which was released in 1834. The report criticized the existing poor legal system and suggested significant changes. Although Chadwick was not selected as one of the three Poor Law Commissioners, he became secretary and wielded influence to advocate for additional Poor Law reforms. However, his insistence on implementing the Act according to his vision and his inability to collaborate with the commissioners created friction. Chadwick's unyielding approach, which left little room for compromise, would prove problematic in future endeavors, including public health initiatives. Typhoid outbreaks in major cities in 1837 and 1838 prompted the government to assign Chadwick to investigate urban sanitation in the UK. In 1842, with assistance from Dr. Thomas Southwood Smith, Chadwick published his groundbreaking report, 'The Sanitary Conditions of the Laboring Population’ [9]. Chadwick's study highlighted that illness and death were not unavoidable results of economic hardship but could be largely prevented through appropriate hygiene measures. He contended that enhancing sewage systems, supplying clean water, and ensuring proper waste disposal would not only improve public health but also decrease the financial strain on society by reducing the need for medical treatment and welfare assistance. The study mobilized public and political backing for hygiene reform, resulting in the creation of local health authorities and the eventual enactment of the Public Health Act of 1848 [10]. The miasma theory of disease, which was widely accepted during that period, further emphasized the necessity for reform. This theory proposes that ailments such as cholera were spread by "foul air" or miasmas originating from decomposing organic matter. Although the germ theory of disease has not yet been fully established, miasma theory has encouraged efforts to clean up urban environments as a method of disease prevention. This belief in the significance of environmental cleanliness set the stage for sanitary awakening and subsequent public health reforms that transformed British cities into more salubrious places to reside in.

The Sanitary Report (1842)

The report on the sanitary conditions of the laboring population, authored by Edwin Chadwick in 1842, is widely regarded as a landmark report in the history of public health. Chadwick's report culminated in his extensive investigation of the deplorable living conditions of Britain's working classes, particularly in rapidly urbanizing areas. The report presented a comprehensive analysis of the relationship between poor living conditions, inadequate sanitation, and the prevalence of diseases among the laboring population. Chadwick collected data on mortality rates, environmental conditions, and the spread of diseases, such as typhus and cholera. His findings emphasized the urgent need for public health reforms and set the stage for sanitary awakening [11]. Chadwick's report was groundbreaking because it explicitly linked the health of the population to environmental factors such as inadequate housing, poor drainage, and contaminated water supplies. He contended that the government was responsible for intervening in and improving these conditions to reduce disease and prevent premature deaths. Chadwick's call for sanitary reform was rooted in both humanitarian and economic concerns. He believed that improving public health would reduce the financial burden on society, as fewer resources would be needed for poor relief and medical care if the population were healthier. The report's detailed recommendations include providing clean water, proper sewage disposal, and establishing local health boards to oversee public health measures [12]. The report indicated that there was an urgent need to ameliorate the living conditions of the impoverished population and that the deficiency in public health was directly correlated with the lifestyles experienced by the poor. Chadwick further observed that the laboring class could not perform as efficiently as required in an expanding industrial economy because of their poverty and poor health. However, the proposed improvements in the report had one significant limitation, their cost, which brought Chadwick into conflict with the numerous influential individuals who were reluctant to expend resources to assist the poor. Chadwick's report focused on the UK's industrial cities, encompassing hundreds of thousands of people. The Conservative government of 1842 effectively rejected Chadwick's report, a situation that persisted until 1847, when a liberal government under Lord John Russell assumed power. Russell demonstrated considerably more sympathy towards the report, and consequently, in 1848, a Public Health Act was enacted.

Advocacy for sanitation

Chadwick, while not only working as a report writer, was also an ardent supporter of sanitation reform. As Secretary of the Poor Law Commission, he used his position to promote the adoption of his ideas. Chadwick believed that controlling the spread of diseases required addressing environmental causes, advocating for the implementation of comprehensive sewer systems, and access to clean water as fundamental measures of public health. He not only recommended policy changes but also actively spoke out in public and lobbied to support sanitation reforms [13]. Chadwick's efforts were instrumental in raising public awareness of the importance of sanitation and preventing diseases by promoting environmental improvements. He emphasized the significance of waste removal and street cleaning as essential aspects of urban sanitation, arguing that these measures would improve health outcomes and the quality of city living. Through persistent lobbying, Chadwick ultimately contributed to the establishment of local health boards that were responsible for implementing urban sanitation measures [14]. In 19th-century England, Edwin Chadwick and John Snow made significant contributions to the advancement of public health. Chadwick's work focused on sanitary reform, notably in his seminal 1842 report. This document elucidates the correlation between poor living conditions, inadequate sanitation, and disease, ultimately resulting in the implementation of the Public Health Act of 1848. Snow, regarded as a pioneer in epidemiology, gained recognition for his groundbreaking research on the 1854 cholera outbreak in London. He successfully identified contaminated water from the Broad Street pump as the source of the disease, effectively demonstrating the critical importance of clean water for cholera prevention. The collective efforts of these two individuals have established a foundation for contemporary public health practices.

Influence on legislation

Chadwick's research has played a critical role in shaping public health policies, particularly the Public Health Act of 1848. This Act, which was instrumental in enhancing public health on a national scale in Britain, established a Central Board of Health, of which Chadwick was a member, and empowered local authorities to implement sanitary measures, such as constructing sewers, providing clean water, and regulating waste disposal [15]. The Act represented a significant departure from the previous disease management approach to a proactive strategy that emphasized prevention. Chadwick's focus on environmental factors as determinants of health was reflected in subsequent legislation and reforms, many of which continued to shape public health policies in Britain and other countries well into the 20th century. Although the Act faced resistance from certain quarters, particularly those who were apprehensive about government intervention in local affairs, it ultimately paved the way for the development of modern public health systems.

Impact of Chadwick’s work on the sanitary awakening

Immediate Impact

Edwin Chadwick's work produced a profound and immediate effect on public health in Britain. His report, "The Report on the Sanitary Condition of the Labouring Population," published in 1842, prompted significant changes that transformed urban sanitation and public health practices. One of the immediate outcomes of Chadwick's efforts was the establishment of local health boards under the Public Health Act of 1848. These health boards were tasked with overseeing the implementation of sanitary measures in cities and towns, such as the construction of sewer systems, provision of clean water, and regulation of waste disposal. The formation of these boards marked the beginning of organized, government-led public health initiatives aimed at improving the living conditions of the urban poor. Chadwick's advocacy for sanitation also led to tangible improvements in urban infrastructure [16]. Cities began to invest in modern sewer systems, which significantly reduced the prevalence of waterborne diseases, such as cholera and typhoid. The emphasis on clean water supply and effective waste management helped decrease the overall burden of disease in urban areas, leading to a measurable improvement in public health. The immediate impact of Chadwick's reforms was most evident in the reduction in mortality rates in cities where sanitary measures were implemented, demonstrating the effectiveness of his approach to public health.

Long-Term Impact

Chadwick's ideas and reforms had a lasting impact, extending beyond Britain's borders. His work was instrumental in initiating a broader sanitary awakening across Europe and laid the basis for the development of contemporary public health systems. The principles advocated by Chadwick, such as the significance of clean water, appropriate sewage disposal, and the government's responsibility for public health, were adopted by other countries and have become a critical aspect of public health policies worldwide [17]. The creation of public health institutions and the professionalization of public health practices reflect Chadwick's influence. He emphasized the importance of data collection, scientific investigation, and government intervention in public health, thus paving the way for the development of epidemiology and public health as scientific disciplines. Over time, the ideas promoted by Chadwick became the foundation of public health systems in Europe and beyond, resulting in the establishment of national public health services and the widespread adoption of sanitary reforms. The enduring effect of Chadwick's work is evident in the sustained improvement in public health following sanitary awakening, particularly in urban areas, where reforms were most rigorously implemented [18].

Legacy of Edwin Chadwick

Edwin Chadwick's influence on public health has been monumental and has significantly contributed to the formation of contemporary public health systems. His groundbreaking work, notably his 1842 "Report on the Sanitary Condition of the Labouring Population," has greatly contributed to the construction of the public health infrastructure we recognize today. Chadwick's report revealed the deplorable living conditions of urban citizens and unequivocally demonstrated a correlation between unsanitary conditions and the spread of disease. This report played a crucial role in advocating extensive public health reforms, which ultimately led to the passage of the Public Health Act of 1848, the first act of its kind in Britain [19]. The Public Health Act of 1848 established the General Board of Health to oversee the implementation of sanitary measures across the country and mandated the provision of clean water, construction of sewers, and regulation of waste disposal, key components of Chadwick's vision for improving public health. These reforms not only helped to reduce the spread of infectious diseases, such as cholera and typhoid but also set a precedent for government intervention in public health that has continued to evolve over the years. Chadwick's influence extended beyond the immediate reforms of the mid-19th century, leaving a lasting impact on public health policies and practices. Chadwick's work has had a profound influence on public health, particularly in sanitary engineering and urban planning. His emphasis on the importance of clean water and effective waste management laid the groundwork for future public health initiatives and significantly affected the development of infrastructure that prioritized public health. His pioneering approach of focusing on preventative measures rather than treating diseases has had a global influence on public health policies. The effects of Chadwick's advocacy can be seen in the work of subsequent public health reformers, who expanded upon his ideas and established public health laws and health boards not only in Britain but also in other industrializing nations. Chadwick's commitment to using scientific methods, such as data and evidence, to inform public health policies has become standard practice in modern public health and epidemiology [20,21].

Accolades and monuments

Sir Edwin Chadwick's influence on public health has been widely recognized and celebrated, despite the controversy surrounding his approach and personality during his lifetime. In 1889, he was knighted as a significant honor that reflected the importance of his contributions to public health. Moreover, several institutions and awards have been named in his honor, such as the prestigious Chadwick Medal awarded by the American Public Health Association to individuals who have made significant contributions to public health [22]. This medal serves as a testament to Chadwick's lasting influence in the field. In addition, Chadwick's legacy is preserved through various public health institutions and initiatives that are guided by the principles he established during the sanitary awakening.

## Conclusions

Edwin Chadwick made substantial contributions to the field of public health, particularly during the sanitary awakening, which significantly improved urban health conditions in the 19th century. His work laid the groundwork for the Public Health Act of 1848 and established fundamental principles of sanitation that remain essential today. Chadwick's emphasis on clean water, sewage systems, and public health infrastructure has had a lasting effect on modern public health policies and practices. His legacy continues to be relevant, emphasizing the importance of preventive health measures in maintaining population health.

## References

[1] Mackenbach JP (1999). Health, civilization and the state: a history of public health from ancient to modern times. BMJ.

[2] Guha S (1994). The importance of social intervention in England's mortality decline: the evidence reviewed. Soc Hist Med.

[3] Hardy A (1999). Public health and social justice in the age of Chadwick: Britain, 1800-1854. Med Hist.

[4] Green MA, Dorling D, Mitchell R (2018). Updating Edwin Chadwick's seminal work on geographical inequalities by occupation. Soc Sci Med.

[5] Halliday S (2001). Death and miasma in Victorian London: an obstinate belief. BMJ.

[6] (2024). National Library of Medicine. Edwin Chadwick: Pioneer of Public Health Reform. http://resource.nlm.nih.gov/101434230.

[7] Small H (2022). Edwin Chadwick: a biographical update. J Med Biogr.

[8] Colgrove J (2002). The McKeown thesis: a historical controversy and its enduring influence. Am J Public Health.

[9] Mushtaq MU (2009). Public health in british India: a brief account of the history of medical services and disease prevention in colonial India. Indian J Community Med.

[10] Thomson K, Hillier-Brown F, Todd A, McNamara C, Huijts T, Bambra C (2018). The effects of public health policies on health inequalities in high-income countries: an umbrella review. BMC Public Health.

[11] Alderslade R (1998). The Public Health Act of 1848. The Act's qualities of imagination and determination are still needed today. BMJ.

[12] Hamlin C (1996). Edwin Chadwick, "mutton medicine," and the fever question. Bull Hist Med.

[13] Abbasi M, Majdzadeh R, Zali A, Karimi A, Akrami F (2018). The evolution of public health ethics frameworks: systematic review of moral values and norms in public health policy. Med Health Care Philos.

[14] Smith RF (2015). Narratives of public health in Dickens's journalism: the trouble with sanitary reform. Lit Med.

[15] Ringen K (1979). Edwin Chadwick, the market ideology, and sanitary reform: on the nature of the 19th-century public health movement. Int J Health Serv.

[16] Golding AM (2006). Sir Edwin Chadwick and inequalities. Public Health.

[17] Corbett SJ (2007). Channelling Edwin Chadwick: beyond utopian thinking in urban planning policy and health. N S W Public Health Bull.

[18] Hanley J (2002). Edwin Chadwick and the poverty of statistics. Med Hist.

[19] Sram I, Ashton J (1998). Millennium report to Sir Edwin Chadwick. BMJ.

[20] Khoury MJ, Gwinn M, Ioannidis JP (2010). The emergence of translational epidemiology: from scientific discovery to population health impact. Am J Epidemiol.

[21] (2016). Revolutions in public health: 1848, and 1998?. BMJ.

[22] Gelfand T (1983). Death is a social disease: public health and political economy in early industrial France. Isis.

